# Identification and validation of immune-related hub genes based on machine learning in prostate cancer and AOX1 is an oxidative stress-related biomarker

**DOI:** 10.3389/fonc.2023.1179212

**Published:** 2023-07-31

**Authors:** Xiaocong Mo, Kaisheng Yuan, Di Hu, Cheng Huang, Juyu Luo, Hang Liu, Yin Li

**Affiliations:** ^1^ Department of Oncology, the First Affiliated Hospital of Jinan University, Jinan University, Guangdong, Guangzhou, China; ^2^ Department of Metabolic and Bariatric Surgery, the First Affiliated Hospital of Jinan University, Jinan University, Guangdong, Guangzhou, China; ^3^ Department of Neurology and Stroke Centre, the First Affiliated Hospital of Jinan University, Jinan University, Guangdong, Guangzhou, China; ^4^ Department of Urology, the First Affiliated Hospital of Chongqing Medical University, Chongqing Medical University, Chongqing, China

**Keywords:** biomarker, prostate cancer, AOX1, CIBERSORT, diagnostic

## Abstract

To investigate potential diagnostic and prognostic biomarkers associated with prostate cancer (PCa), we obtained gene expression data from six datasets in the Gene Expression Omnibus (GEO) database. The datasets included 127 PCa cases and 52 normal controls. We filtered for differentially expressed genes (DEGs) and identified candidate PCa biomarkers using a least absolute shrinkage and selector operation (LASSO) regression model and support vector machine recursive feature elimination (SVM-RFE) analyses. A difference analysis was conducted on these genes in the test group. The discriminating ability of the train group was determined using the area under the receiver operating characteristic curve (AUC) value, with hub genes defined as those having an AUC greater than 85%. The expression levels and diagnostic utility of the biomarkers in PCa were further confirmed in the GSE69223 and GSE71016 datasets. Finally, the invasion of cells per sample was assessed using the CIBERSORT algorithm and the ESTIMATE technique. The possible prostate cancer (PCa) diagnostic biomarkers AOX1, APOC1, ARMCX1, FLRT3, GSTM2, and HPN were identified and validated using the GSE69223 and GSE71016 datasets. Among these biomarkers, AOX1 was found to be associated with oxidative stress and could potentially serve as a prognostic biomarker. Experimental validations showed that AOX1 expression was low in PCa cell lines. Overexpression of AOX1 significantly reduced the proliferation and migration of PCa cells, suggesting that the anti-tumor effect of AOX1 may be attributed to its impact on oxidative stress. Our study employed a comprehensive approach to identify PCa biomarkers and investigate the role of cell infiltration in PCa.

## 1 Introduction

Prostate cancer (PCa) is a significant global health concern, with 1.6 million cases and 366,000 deaths occurring worldwide each year. It ranks as the second most common cancer globally and the fifth leading cause of male mortality (1). Due to its biological characteristics, distant micrometastases, and localized residuals, PCa has an increasing likelihood of recurrence. However, there are certain curative therapies available, such as radical prostatectomy (RP). When utilized early during recurrence, salvage therapy can effectively decrease the risk of distant metastases, prolong lifespan, and potentially lead to a cure. Therefore, the early detection of PCa plays a vital role in improving prognosis and reducing patient mortality (2).

Historically, PCa has been diagnosed using a blood test for prostate-specific antigen (PSA), a digital rectal examination (DRE), and a prostate biopsy. However, PSA lacks specificity, which leads to the over-diagnosis and overtreatment of PCa. As a result, there is a growing clinical need for the identification of new biomarkers that can serve as prognostic, predictive, and therapeutic response indicators. These biomarkers can be utilized to implement a precision medicine strategy for the management of PCa (3). For instance, studies have demonstrated that the deletion of phosphatase and tensin homolog (PTEN) is associated with a poor prognosis in PCa patients (4). The loss of PTEN in biopsy samples has been shown to predict an increased risk of castration-resistant prostate cancer (CRPC), metastasis, and PCa-specific mortality (5, 6). To analyze the molecular processes and genomic effects of the co-deletion of BRCA2 and RB1 in PCa, previous research has shown that the deletion of BRCA2 leads to a castration-resistant phenotype in human PCa cell lines (LNCaP and lapc4) (7). This suggests that it is possible to investigate the molecular pathways involved in the progression of PCa and explore new diagnostic approaches for this disease. However, to date, no study has combined the least absolute shrinkage and selector operation (LASSO) regression model with support vector machine recursive feature elimination (SVM-RFE) to identify PCa biomarkers.

In recent years, immunology research has shown that immune cell infiltration plays a crucial role in the development and progression of PCa. For instance, Flammiger et al. conducted a study on prostate cancer specimens and used forkhead box P3 (FOXP3) immunohistochemistry to detect regulatory T cells (Tregs). They found that the increase in Tregs was associated with an advanced and worsening prognosis in prostate cancer tissues (8). Additionally, Eastham et al. compared normal patients with PCa patients and observed that the level of transforming growth factor-beta (TGF-β) was higher in PCa patients. This increase in TGF-β promoted both migration and invasion of PCa cells (9). Tissue microarray analysis confirmed decreased levels of FOXA1 protein and increased TGF-β signaling pathway in castration-resistant prostate cancer (CRPC) compared to primary tumors, which suppresses CRPC progression (10). Furthermore, some studies have demonstrated that tyrosine hydroxylase 2 (Th2) and central memory T cell (TCM) are associated with prostate cancer recurrence after radical prostatectomy (RP) and act as independent protective factors (11). However, so far, only a limited number of studies have utilized the CIBERSORT technique to investigate the infiltration of immune cells and potential biomarkers in prostate cancer.

We obtained six publicly available datasets on PCa from the Gene Expression Omnibus (GEO) database. To create a metadata cohort, we combined four of these datasets and used them as the training group. The remaining two datasets were merged into another metadata cohort, which served as the treatment group. Within the training group, we compared 127 PCa cases with 52 normal controls to identify differentially expressed genes (DEGs). Machine-learning techniques were then employed to screen and identify diagnostic biomarkers for PCa. These candidate genes were subsequently validated in the treatment group. Additionally, the CIBERSORT methods were used to examine the correlation between biomarkers and immune cells infiltrating PCa. This analysis aimed to enhance our understanding of the molecular immunological processes involved in PCa and establish a practical and conceptual framework for future research.

## 2 Methods and materials

### 2.1 Gene expression data acquisition and processing

We utilized the GEO database to gather information on PCa. Specifically, we downloaded raw data from the GSE8511, GSE14206, GSE46602, GSE55945, GSE69223, and GSE71016 datasets. These datasets were then divided into two groups: the training group (consisting of GSE8511, GSE14206, GSE46602, and GSE55945) with 127 PCa cases and 52 normal controls, and the test group (consisting of GSE69223 and GSE71016) with 63 PCa cases and 62 normal controls. To ensure consistency and eliminate any potential biases, we merged the datasets within each group and applied preprocessing techniques, including the use of the ‘SVA’ package’s combat capabilities to remove any batch effects (12) (**Table 1**).

**Table 1:** Information for selected GEO datasets.

GEO accession	country	Platform	Samples		Category
			PCa	Normal	
GSE8511	USA	GPL1708	24	16	Train group
GSE14206	Italy	GPL887	53	14	Train group
GSE46602	Denmark	GPL570	36	14	Train group
GSE55945	USA	GPL570	13	8	Train group
GSE69223	Germany	GPL570	15	15	Test group
GSE71016	USA	GPL16699	48	47	Test group

### 2.2 Identification of DEGs in PCa

The R package ‘limma’ from http://www.bioconductor.org/ was used to detect differentially expressed genes (DEGs) between 127 PCa patients and 52 normal controls in the training group (13). DEGs were identified based on a threshold of |log fold change (FC)| > 1 and an adjusted false discovery rate (*P* < 0.05). The comparison was made between 127 PCa cases and 52 normal controls in the training group. The volcano plot was generated using the R software package ‘ggplot2’ to visualize the DEGs. Additionally, a heat map of the DEGs was created using the R package ‘heatmap’.

### 2.3 Functional enrichment analysis

Gene module-related functions were identified through functional enrichment studies conducted using the R package ‘cluster profile’. These studies utilized the gene ontology (GO), the disease ontology (DO) ontologies, and the Kyoto encyclopedia of genes and genomes (KEGG) (14). To perform gene set enrichment analysis (GSEA) on the training group, we examined signal pathway differences. GSEA analysis was conducted on the gene expression matrix using the ‘cluster Profiler’ and ‘enrich plot’ programs, with the reference gene set as ‘c2.cp.kegg.v7.4.symbols.gmt’ (15). KEGG GSEA analysis was separately performed on the PCa and normal cases of the training group. Significant saturation was defined as *P* < 0.05.

### 2.4 Screening characteristic related biomarkers *via* machine learning

Two machine learning methods were utilized to evaluate potential prognostic factors in prostate cancer (PCa). The LASSO technique, implemented with the R package ‘glmnet’, was employed to identify genes that were significantly associated with the differentiation between PCa and normal patients (16). Additionally, the support vector machine (SVM) was utilized as a surveillance machine learning technique to identify the optimal variables by eliminating feature vectors. To mitigate overfitting, a recursive feature elimination (RFE) approach was used to select the best genes. Therefore, SVM-RFE was employed to determine the gene set with the highest discriminatory power. To conduct classification analysis on the selected biomarkers for PCa diagnosis, we utilized the SVM-RFE classifier from the R package ‘e1071’ (17). Subsequently, a Venn diagram was employed to identify the overlapping genes obtained from both algorithms. These genes will be further validated in the test group.

### 2.5 Diagnostic value of the biomarkers in PCa

To compare the differences of these genes in the test group and assess the predictive value of established biomarkers, we utilized the R package ‘ggpubr’. A significance level of *P* < 0.05 was considered statistically significant (18). Subsequently, we employed the R package ‘proc’ to generate an ROC curve in the training group consisting of 127 PCa cases and 52 normal controls (19). Hub genes were defined as those with a value greater than 85% (AUC). The diagnostic impact of PCa on normal samples was evaluated by calculating the AUC value, which was then verified in the test group comprising 63 PCa cases and 62 normal controls.

### 2.6 Assessment of immune cell infiltration

The CIBERSORT method was used to classify 22 different kinds of immune cell matrix. A reference set of 22 immune cell subtypes was utilized to assess the presumed abundance of immune cells, with 1,000 permutations (20). The invasion of the immune cell matrix was generated based on a significance level of *P* < 0.05. The program ‘corrplot’ was used to illustrate the association within 22 different kinds of immune cell infiltration and to create a correlation between heatmap and boxplot (21). Violin plots were created using the R package ‘vioplot’ to illustrate the variations in the infiltration of immune cells between PCa and normal samples (22).

### 2.7 Analysis of correlations between identified genes and immune cell infiltration

We conducted a Spearman’s rank correlation analysis using R software to examine the correlation between the levels of expression of the identified biomarkers and the level of infiltrating immune cells (23). The resulting correlations were visualized using the charting approach provided by the ‘ggpubr’ package (24).

### 2.8 Cell culture and transfection

The PCa cell lines (LNCaP, PC3, and DU145) and the normal prostate cell line (RWPE-1) were cultured in RPMI-1640 medium. Oe-AOX1 and its negative control (Oe-ctrl) were synthesized by GenePharma (Shanghai, China). LNCaP and PC3 cells were evenly plated in 24-well plates. Once the two cell lines reached approximately 80-90% confluence, they were transfected following the provided instructions.

### 2.9 Western blot analysis

Cell proteins were separated by electrophoresis on a 12% SDS-PAGE gel and then transferred to PVDF membranes. The membranes were blocked with a 5% solution of silk milk at room temperature for 1 hour. Subsequently, the membranes were incubated overnight at 4°C with primary antibodies against AOX1 (ab92519; 1:500; Abcam; USA) and β-actin (ab8226; 1:2,500; Abcam; USA), followed by incubation at room temperature for 1 hour with secondary antibodies (ab6721; 1:3,000; ab6728; 1:3,000; Abcam; USA). The membranes were then visualized using an enhanced ECL detection kit (Beyotime, China).

### 2.10 RT-qPCR analysis

RNA was extracted from PCa cells using TriZol (Beyotime, China). The extracted RNA was then reverse transcribed into complementary DNA. The quantified expressions were detected using SYBR Green qPCR Master Mix and the 2^-ΔΔCq^ method.

### 2.11 Cell proliferation assay

Ninety-six well plates were used to seed PCa cells (LNCaP and PC3). The plates were then incubated at 37°C and 5% CO_2_. After incubation, a CCK-8 reagent test kit (Tiangen) was added at a volume of 10 µl per well. The PCa cells were further incubated at 37°C and 5% CO2 for 1 hour. Finally, the optical density (OD) value at 450nm was measured using a microplate reader for analysis.

### 2.12 Clone formation assay

The cultured cells in the logarithmic growth phase were diluted and seeded into dishes containing culture medium at the appropriate gradient density. The cells were then cultured at 37°C with 5% CO_2_ for a period of 2 weeks. Afterward, the cells were washed twice with PBS and fixed with paraformaldehyde for 15 minutes. Subsequently, the colonies were stained with 0.1% crystal violet for 15 minutes and washed off with water. Finally, clones consisting of more than 10 cells were counted.

### 2.13 Measurement of malondialdehyde

Cells were lysed and centrifuged at 10,000g for 10 minutes. The MDA content of the cells was measured using the MDA Assay Kit (S0131, Beyotime, China). The samples were tested at 532 nm using a microplate reader and compared to the standard curve of MDA.

### 2.14 Measurement of Glutathione and ROS

The contents of GSH and ROS were detected using the corresponding kits, following the reference protocols provided by the manufacturer.

### 2.15 Statistical analysis

All statistical analyses were performed using Perl version 5.32.1 and R software version 4.1.2. P < 0.05 was used to determine statistical significance.

## 3 Results

### 3.1 Identification of DEGs in PCa

**Figure 1:**
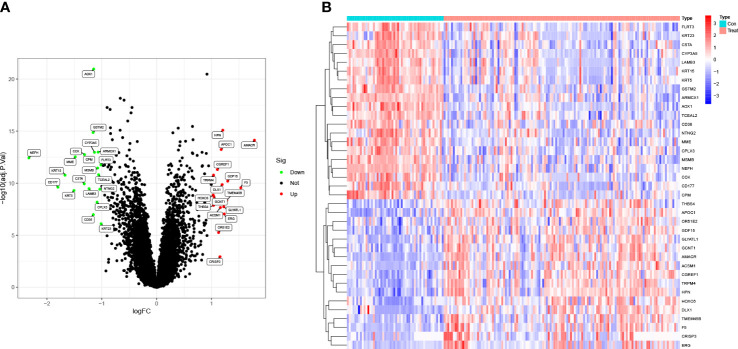
Identification of DEGs in PCa. **(A)** The volcano plot showed that 17 upregulated genes (red) and 20 downregulated genes (green) from 127 PCa cases and 52 normal controls in GEO datasets (*P*<0.05). Black dots mean meaningless (*P*>0.05). **(B)** The heat map showed the expression levels of 37 DEGs.

The differentially expressed genes (DEGs) from the GEO databases (GSE8511, GSE14206, GSE46602, and GSE55945) in 127 PCa cases and 52 normal controls were identified using the R package ‘limma.’ Out of the 37 DEGs, 17 were up-regulated and 20 were down-regulated. A log fold change (FC) > 0 indicates up-regulation in the training group, while a log FC < 0 indicates down-regulation. These findings are visually represented in the volcano plot (**Figure 1A**). The expression levels of the 37 DEGs are further illustrated in the heat map (**Figure 1B**).

### 3.2 Functional enrichment analysis

**Figure 2:**
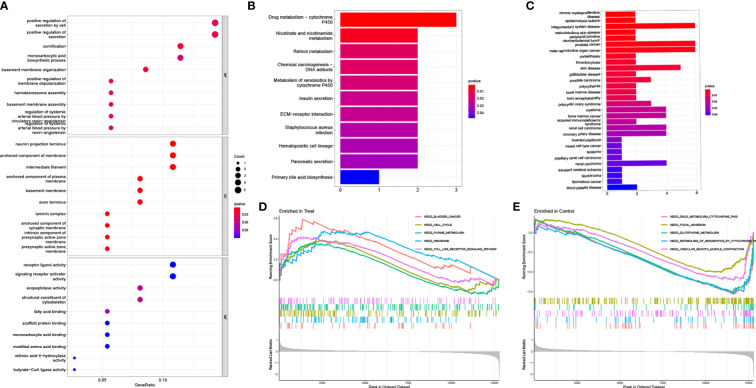
Functional enrichment analysis. **(A)** GO enrichment analysis, **(B)** KEGG enrichment analysis and **(C)** DO enrichment analysis of DEGs between PCa and control samples in train group. Enrichment analyses *via* gene set enrichment analysis in **(D)** PCa patients and **(E)** control samples of train group, respectively.

To evaluate the probable biological activities of the 37 DEGs, we conducted GO, KEGG, DO, and GSEA analyses using the R package ‘cluster profile’. The GO results revealed that the majority of these genes were associated with basement membrane organization, cornification, and positive regulation of secretion by cells (**Figure 2A**). KEGG enrichment analysis identified genes involved in drug metabolism, specifically cytochrome P450, nicotinate and nicotinamide metabolism, and retinol metabolism (**Figure 2B**). The results from the DO analysis revealed that the diseases enriched by DEGs were primarily associated with chronic myeloproliferative diseases, epidermolysis bullosa, integumentary system disease, vesiculobullous skin disease, peripheral primitive neuroectodermal tumor, and prostate cancer (**Figure 2C**). In the PCa group, the GSEA results demonstrated that the enriched pathways mainly included bladder cancer, cell cycle, purine metabolism, ribosome, and toll-like receptor signaling pathway (**Figure 2D**). On the other hand, in the control group, the GSEA results showed that the enriched pathways mainly involved glutathione metabolism, focal adhesion, cytochrome P4 metabolism of xenobiotics, cytochrome P450 metabolism of drugs, and vascular smooth muscle contraction (**Figure 2E**).

### 3.3 Screening diagnostic feature biomarkers for PCa

**Figure 3:**
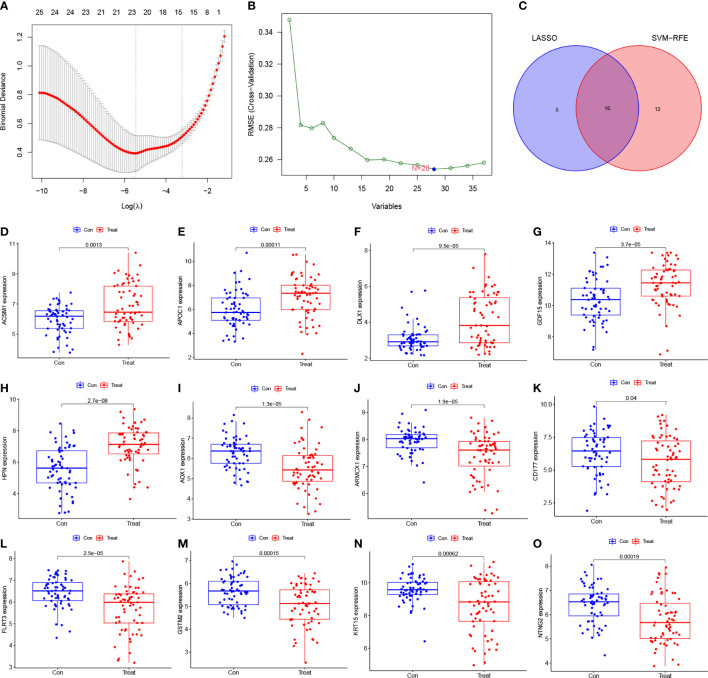
Screening diagnostic feature biomarkers for PCa. **(A)** Twenty-one genes were identified by LASSO regression. **(B)** A plot of biomarkers selection *via* SVM-RFE algorithm. **(C)** Venn diagram demonstrating sixteen diagnostic markers shared by the LASSO and SVM-RFE algorithms. **(D-O)** Validation of the expression levels of PCa-related diagnostic biomarkers in the test group (all *P* < 0.05), including **(D)** ACSM1; **(E)** APOC1; **(F)** DLX1; **(G)** GDF15; **(H)** HPN; **(I)** AOX1; **(J)** ARMCX1; **(K)** CD177; **(L)** FLRT3; **(M)** GSTM2; **(N)** KRT15; **(O)** NTNG2. LASSO, least absolute shrinkage and selection operator; SVM, support vector machine; RFE, recursive feature elimination.

To identify potential diagnostic biomarkers, we employed two distinct approaches. Firstly, we utilized the LASSO logistic regression approach to detect twenty-one genes as potential biomarkers for PCa from the robust DEGs (**Figure 3A**). Secondly, we employed the SVM-RFE technique to determine 28 genes from the DEGs (**Figure 3B**). Finally, we employed the Venn diagram to identify the overlapping gene markers obtained from both methods. As a result, we obtained sixteen related biomarkers, namely AOX1, HPN, GSTM2, APOC1, ARMCX1, FLRT3, MSMB, KRT15, GDF15, DLX1, CD177, NTNG2, CPLX3, ACSM1, ERG, and CD38 (**Figure 3C**).

### 3.4 Identification and validation of diagnostic feature biomarkers for PCa

**Figure 4:**
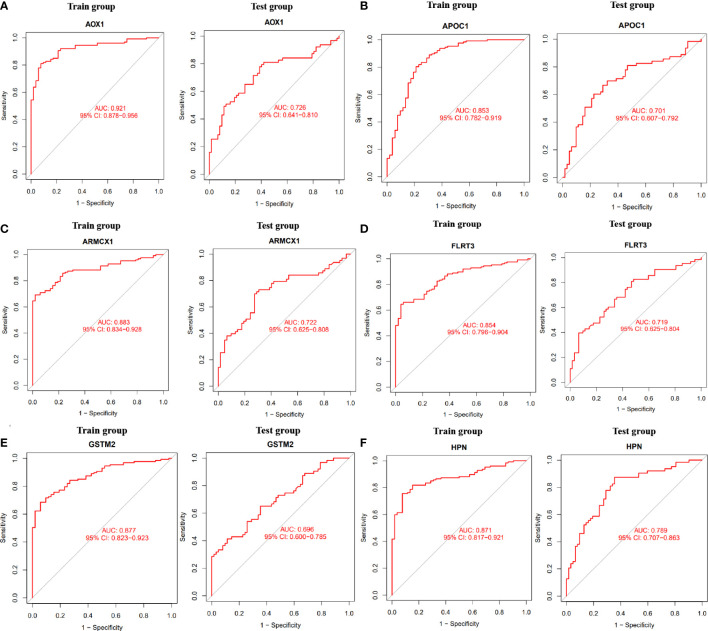
The ROC curve of the diagnostic effectiveness of the six diagnostic markers in the train group and test group. **(A)** AOX1; **(B)** APOC1; **(C)** ARMCX1; **(D)** FLRT3; **(E)** GSTM2; **(F)** HPN.

To determine the expression levels of six genes, ACSM1, APOC1, DLX1, GDF15, HPN, AOX1, ARMCX1, CD177, FLRT3, GSTM2, KRT15, and NTNG2, we utilized the GSE69223 dataset and GSE71016 dataset. Our findings revealed that the expression levels of ACSM1, APOC1, DLX1, GDF15, and HPN were significantly higher in PCa tissues compared to normal tissues (**Figures 3D–H**); all *P* < 0.05). On the other hand, the opposite outcome was seen for AOX1, ARMCX1, CD177, FLRT3, GSTM2, KRT15, 233 and NTNG2 (**Figures 3I–O**); all *P* < 0.05). Additionally, there was no significant change in the amounts of CD38, CPLX3, ERG, and MSMB between PCa tissues and the normal tissue (all *P* > 0.05). In the training group, we constructed ROC curves for these twelve genes, defining hub genes as those with an AUC greater than 85%. Then, we identified six PCa-related diagnostic genes, and the AUC of AOX1 was 0.921 (95% CI 0.878-0.956), APOC1 was 0.853 (95% CI 0.782-0.919), ARMCX1 was 0.883 (95% CI 0.834-0.928), FLRT3 was 0.854 (95%CI 0.796-0.904), GSTM2 was 0.877 (95%CI 0.823-0.923) and HPN was 0.871 (95%CI 0.817-0.921).Then, a robust discrimination was proved in the GSE69223 and GSE71016 datasets, and the AUC of AOX1 was 0.726 (95% CI 0.641-0.810), APOC1 was 0.701 (95% CI 0.607-0.792), ARMCX1 was 0.722 (95% CI 0.625-0.808), FLRT3 was 0.719 (95% CI 0.625-0.804), GSTM2 was 0.696 (95% CI 0.600-0.785) and HPN was 0.789 (95%CI 0.707-0.863) (**Figures 4A–F**).

### 3.5 Assessment of immune cell infiltration

**Figure 5:**
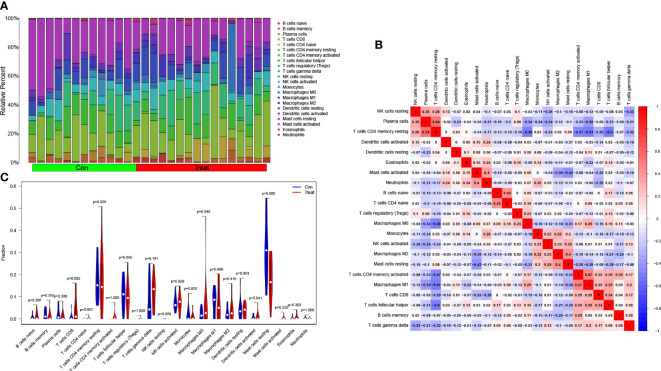
Assessment of immune cell infiltration between PCa and normal controls. **(A)** The box-plot diagram indicating the relative proportions of 22 different immune cell subtypes between PCa and normal controls; **(B)** The heat map shows the correlation among 22 different immune cell populations, with red and blue corresponding to positive and negative correlations, respectively. White indicates an absence of any correlation between the indicated immune cell populations; **(C)** The difference of immune infiltration between PCa (red) and normal (blue) controls (*P*<0.05 was regarded as statistically significant).

Following that, we utilized the CIBERSORT technique to visualize the invasion of 22 distinct immune cell kinds in the training group (**Figure 5A**). Additionally, the CIBERSORT method demonstrated the invasion of 22 distinct immune cell types, and the heatmaps showed strong positive relationships between T cells CD4 memory resting and plasma cells (r=0.54) and strong inverse relationships between T cells CD4 memory resting and macrophages M1 (r= -0.51) (**Figure 5B**). We studied the component of immune cells in PCa and normal tissues. T cells CD8 in PCa was remarkably higher compared with the normal controls as indicated in the findings (*P* = 0.032), while mast cells resting was lower than the normal controls (*P* = 0.005; **Figure 5C**).

### 3.6 Correlation analysis between PCa-related biomarkers and immune infiltrating cells

**Figure 6:**
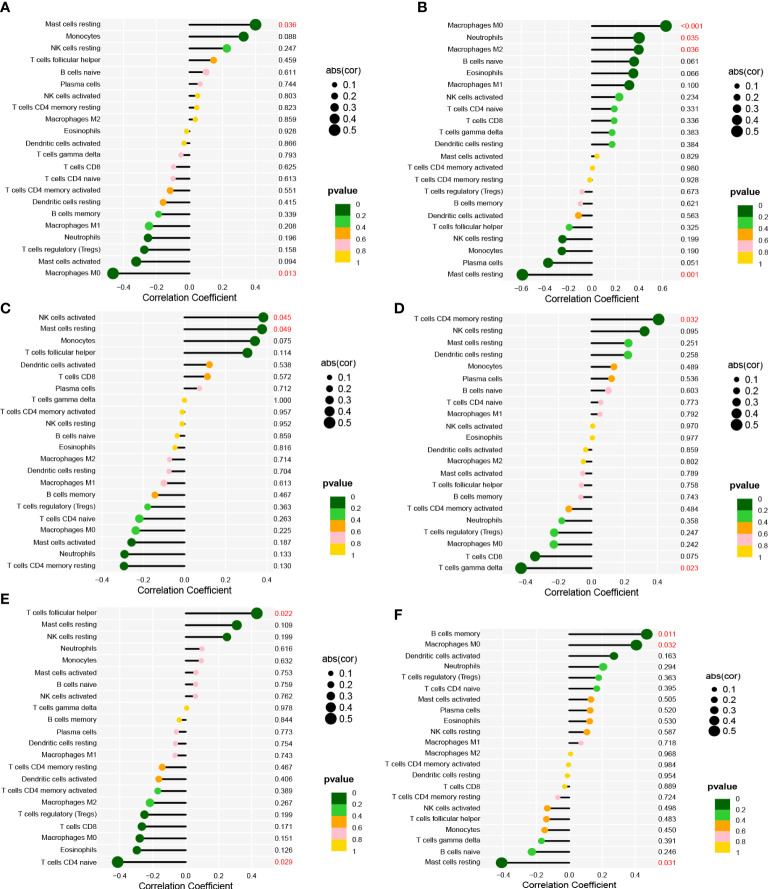
Correlation between **(A)** AOX1; **(B)** APOC1; **(C)** ARMCX1; **(D)** FLRT3; **(E)** GSTM2; **(F)** HPN and infiltrating immune cells in PCa.

The correlation analysis revealed that AOX1 was positively associated with mast cells resting (R = 0.4, *P* = 0.036) and negatively associated with macrophages M0 (R = -0.46, *P* = 0.013; **Figure 6A**). APOC1 was positively associated with macrophages M0 (R = 0.63, *P* < 0.001), neutrophils (R = 0.4, *P* = 0.035), and macrophages M2(R = 0.4, *P* = 0.036) and negatively associated with mast cells resting (R = -0.59, *P* = 0.001; **Figure 6B**). ARMCX1 was positively associated with NK cells activated (R = 0.38, *P* = 0.045) and mast cells resting (R = 0.38, *P* = 0.049; **Figure 6C**). FLRT3 was positively associated with T cells CD4 memory resting (R = 0.41, *P* = 0.032) and negatively associated with T cells gamma delta (R = -0.43, *P* = 0.023; **Figure 6D**). GSTM2 was positively associated with T cells follicular helper (R = 0.43, *P* = 0.022) and negatively associated with T cells CD4 naive (R = -0.41, *P* = 0.029; **Figure 6E**). HPN was positively associated with B cells memory (R = 0.47, *P* = 0.011) and macrophages M0 (R = 0.41, *P* = 0.032) and negatively associated with mast cells resting (R = - 0.41, *P* = 0.031; **Figure 6F**).

### 3.7 Experimental verification of AOX1 in PCa

**Figure 7:**
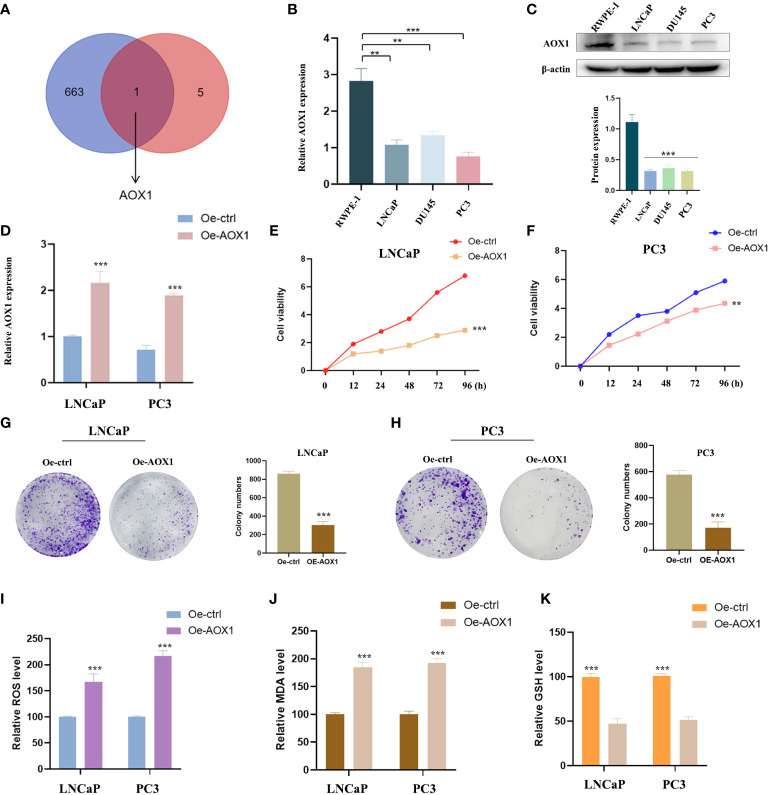
Experimental verification of AOX1 in PCa. **(A)** AOX1 was classified as an oxidative stress gene. **(B, C)** Both mRNA level and protein level showed AOX1 in PCa cell lines expressed higher than those in normal prostate cell line. **(D)** The overexpression transfection was clearly successful in PCa cell lines. **(E, F)** CCK-8 reveled that overexpression of AOX1 could significantly inhibit the proliferation activity of LNCaP and PC3 cell lines. **(G, H)** The clone capacity of LNCaP and PC3 cells were inhibited by the overexpression of AOX1. **(I-K)** Overexpression of AOX1 showed a significant increase in ROS and MDA levels, while an obvious decrease in GSH level. **P <0.01; ***P <0.001.

Oxidative stress is closely related to cancer. To further identify whether these six PCa-related diagnostic genes are related to oxidative stress, we downloaded the gene sets of oxidative stress genes from the website GeneCards (https://www.genecards.org/). After taking the intersection, only AOX1 among the DEGs was classified as an oxidative stress gene (**Figure 7A**). The expression of AOX1 was verified. We obtained several PCa cell lines (LNCaP, PC3 and DU145) for experimental validation, with normal prostate cell line (RWPE-1) as the ctrol group. In **Figures 7B**, **C**, not only mRNA level but also protein level showed the same significant decrease (*P* < 0.05) trend of AOX1 in PCa cell lines. Subsequently, in order to detect the specific role of AOX1 in the progression of PCa, we applied the functional overexpression (oe-AOX1) into PCa cell lines (LNCaP and PC3). **Figure 7D** showed that the overexpression transfection was clearly successful in PCa cell lines. CCK-8 detection reveled that overexpression of AOX1 could significantly inhibit the proliferation activity of LNCaP and PC3 cells (**Figures 7E, F**). Similarly, the colony formation assays clearly revealed that the clone capacity of LNCaP and PC3 cells were inhibited by the overexpression of AOX1 (**Figures 7G, H**). Moreover, we detected the levels of MDA, ROS, and GSH in LNCaP and PC3 cells. The results showed a significant increase in ROS and MDA levels, while an obviously decrease in GSH level (**Figures 7I–K**). To sum up, AOX1 acted as the role of cancer suppressor during the progression of PCa, which may be partly achieved by triggering oxidative stress.

## 4 Discussion

Nowadays, PCa continues to be one of the leading causes of cancer-related deaths in males. Considering the recent achievements of immunotherapy in various hematological and solid malignancies, there is a growing interest in investigating its potential in the treatment of PCa (25). An increasing number of researchers are acknowledging the connection between immune cell infiltration and various diseases, including cancer (26). As a result, immunotherapy is being considered as a potential approach to combat PCa. The CIBERSORT technique has been effectively utilized to determine the presence of immune cells within tumors and assess their impact on the prognosis of gastric cancer, colorectal cancer, breast cancer, and osteosarcoma (20, 27–29). The importance of immune cell infiltration in PCa has not yet been fully understood. The objective of this study was to investigate the significance of immune cell infiltration in PCa and identify potential diagnostic biomarkers.

To the best of our knowledge, this is the first retrospective study to use the combination of the LASSO and RVM-RFE algorithms, along with the CIBERSORT algorithm, to analyze immune cell invasion in PCa. We obtained six datasets from the GEO database, with two datasets merged for the test group and the remaining four datasets merged for the training group. In the training group, we identified a total of 37 differentially expressed genes (DEGs), with 17 genes being up-regulated and 20 genes being downregulated. The results of the gene set enrichment analysis (GSEA) in PCa cases revealed that the enriched pathways primarily involved bladder cancer, cell cycle, purine metabolism, ribosome, and toll-like receptor signaling pathway.

Using two algorithms, we selected sixteen genes as potential PCa-related biomarkers based on their robust differential expression. Subsequently, we analyzed the differences among these sixteen genes in the test group and found that twelve genes showed statistical significance (*P* < 0.05). Finally, we constructed ROC curves for these twelve genes and identified six final PCa-related diagnostic biomarkers (AOX1, APOC1, ARMCX1, FLRT3, GSTM2, and HPN). To evaluate the predictive efficacy of these six diagnostic biomarkers, we computed their ROC curves in the test group.

Additionally, we used CIBERSORT to assess immune cell infiltration in PCa and investigate its role in the disease. It has been observed that an increase in CD8 T cell infiltration and a decrease in mast cell infiltration during rest are associated with the occurrence and progression of PCa. Correlation analysis between biomarkers associated with PCa and immune invading cells revealed significant associations between AOX1, APOC1, ARMCX1, GSTM2, and HPN with resting mast cells. Furthermore, HPN, AOX1, and APOC1 showed significant correlations with macrophages M0. In a study by Florent et al., immunohistochemistry was performed on tumors from 51 patients with node-positive PCa. The presence of a large density of CD8 + T cells in tumors was discovered to be related to an increased risk of clinical progression in patients with node-positive PCa (30). Mast cells are implicated in various disorders, such as hypersensitivity, inflammation, and fibrosis. It is worth noting that mast cells also play a crucial role in tumor progression. In this study, the CIBERSORT algorithm was employed to analyze 52 normal prostate tissues and 497 primary tumors of patients with prostate cancer (PCa) from TCGA. The results revealed a significant difference in the fraction of static mast cells between PCa and normal tissues. Moreover, an increased number of resting mast cells is associated with a poor prognosis. It is important to consider that radiotherapy and targeted molecular treatments may impact the infiltration of resting mast cells in the immune system (31). Somaiyeh et al. (year) conducted a study where they investigated the protective effect of M0 macrophages and THP-1 cells treated with toll-like receptor 4 (TLR4) agonists on etoposide-induced apoptosis in PCa cells. They cultured these cells with the supernatant of P human prostate cancer cell line (PC3) cells and analyzed the results using enzyme-linked immunosorbent assay with flow cytometry (ELISA) (32). Recent studies, including our own findings, suggest that various types of invasive immune cells play a significant role in PCa and should be the focus of future research.

Additionally, emerging evidence has shown a close relationship between oxidative stress and the development and progression of cancer (33, 34). In this study, we examined the gene AOX1 in relation to 6 hub genes and 664 oxidative stress-related genes. Xiong et al. have previously reported that AOX1 is downregulated and functions as a tumor suppressor gene in clear cell Renal Cell Carcinoma (ccRCC) and PCa (35, 36). In this study, we demonstrated that AOX1 expression was reduced in PCa cells. We then conducted functional experiments by transfecting oe-AOX1, which showed that the overexpression of AOX1 inhibited the proliferation and migration of PCa cells, consistent with previous findings. Notably, AOX1 overexpression led to the accumulation of reactive oxygen species (ROS) and malondialdehyde (MDA), while also restoring the glutathione (GSH) content. Overall, our results suggest that the anti-cancer effect of AOX1 may be mediated through the activation of oxidative stress.

The investigation has several limitations that should be considered. Firstly, the sample size of the published datasets is small, which means that our findings need to be validated in larger datasets and clinical trials to determine whether AOX1, APOC1, armcx1, FLRT3, gstm2, and HPN can be used as biomarkers of PCa. Additionally, the CIBERSORT algorithm used in our study was based on limited retrospective gene data. While some earlier studies have found similar results to ours, the analysis of immune cell infiltration in PCa is currently limited, and our conclusion should be verified by a prospective study with a larger sample size. Moreover, We have conducted initial research on the anti-oncogenic role of AOX1 in the malignant progression of PCa. We have also identified a potential mechanism, which involves triggering oxidative stress *in vitro*. However, further rigorous testing is required for thorough verification.

## 5 Conclusions

In our study, we identified AOX1, APOC1, ARMCX1, FLRT3, GSTM2, and HPN as biomarkers associated with prostate cancer (PCa). Further research should focus on investigating the relationship between PCa and immune cell infiltration to enhance the effectiveness of immunomodulatory treatments for PCa patients. Moreover, we conducted experimental validation and discovered that AOX1 functions as a tumor suppressor in PCa by inducing oxidative stress. This finding not only contributes to a better understanding of the pathogenesis of PCa but also opens up new possibilities for clinical treatment.

## Data availability statement

The datasets supporting the conclusions of this article are available in the GEO database, [unique persistent identifier andhyperlink to datasets in http://www.ncbi.nlm.nih.gov/geo]. The datasets generated during and analyzed during the current studyare available from the corresponding author on reasonable request.

## Ethics statement

The study was approved by the Ethics Committee of the First Affiliated Hospital of Jinan University, China.

## Author contributions

XM, KY, DH and YL conceived and designed the experiments; XM, KY, DH and JL conducted the research; XM, DH, YL, KY and HL contributed materials and analysis tools; XM, DH, JL, KY and CH analyzed the results; XM, KY and DH wrote the paper. All authors reviewed the manuscript. XM, KY and DH contributed equally to this work. YL is the corresponding author.
